# The Regulation of Adipose Tissue Health by Estrogens

**DOI:** 10.3389/fendo.2022.889923

**Published:** 2022-05-26

**Authors:** Benjamin M. Steiner, Daniel C. Berry

**Affiliations:** Division of Nutritional Sciences, Cornell University, Ithaca, NY, United States

**Keywords:** white adipose tissue, estrogen, adipocyte progenitor cells, estrogen receptor, metabolically healthy, hypertrophy, adipokines, ovariectomy

## Abstract

Obesity and its’ associated metabolic diseases such as type 2 diabetes and cardiometabolic disorders are significant health problems confronting many countries. A major driver for developing obesity and metabolic dysfunction is the uncontrolled expansion of white adipose tissue (WAT). Specifically, the pathophysiological expansion of visceral WAT is often associated with metabolic dysfunction due to changes in adipokine secretion profiles, reduced vascularization, increased fibrosis, and enrichment of pro-inflammatory immune cells. A critical determinate of body fat distribution and WAT health is the sex steroid estrogen. The bioavailability of estrogen appears to favor metabolically healthy subcutaneous fat over visceral fat growth while protecting against changes in metabolic dysfunction. Our review will focus on the role of estrogen on body fat partitioning, WAT homeostasis, adipogenesis, adipocyte progenitor cell (APC) function, and thermogenesis to control WAT health and systemic metabolism.

## Introduction

**Figure 1:**
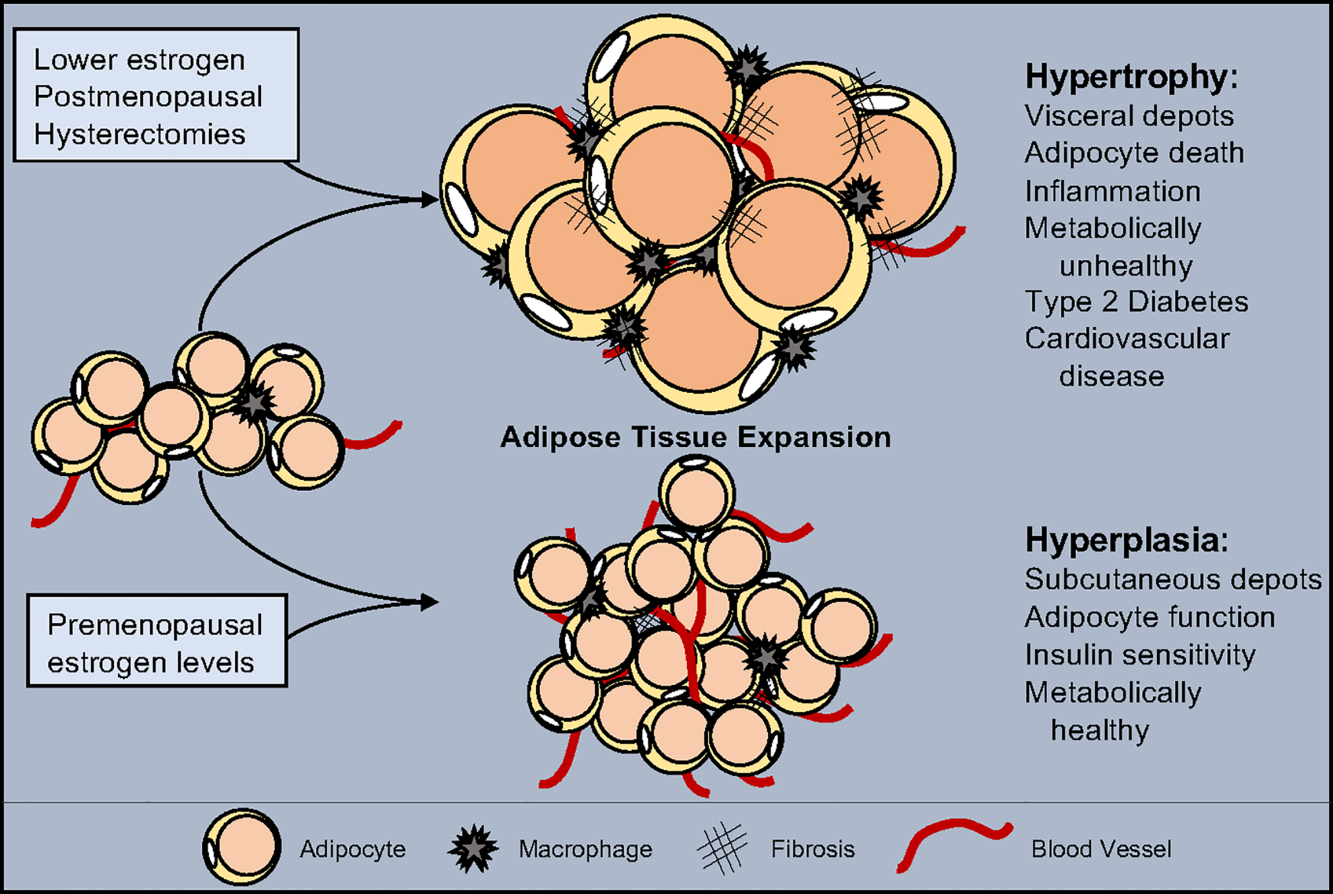
Estrogen influence on adipose tissue expansion. White adipose tissue can expand through either hypertrophy, the swelling of individual adipocytes, or hyperplasia, the increase of adipocyte numbers. Hypertrophy is accompanied by a reduction in vascularization, increased inflammation, and promotes fibrosis. Hyperplasia maintains tissue health by facilitating vascularization, promoting anti-inflammatory signals and block fibrosis. Estrogen levels play a role in determining the type of expansion and location which shifts as estrogen decreases due to pharmacological drugs, medical procedures, and/or age. While normal estrogen levels result in hyperplastic subcutaneous adipose tissue growth, reduced estrogen leads to metabolically unhealthy hypertrophic visceral adipose tissue expansion.

Obesity is a global public health problem (1). Not only is obesity characterized as having excess body fat, but it is also associated with or is a risk factor for developing metabolic dysregulation and cardiometabolic diseases such as insulin resistance, type 2 diabetes, hypertension, arterial cardiovascular disease, and cancer (2–6). These comorbidities occur because white adipose tissue (WAT) is no longer considered an inert storage depot for excess energy but rather an active endocrine organ that regulates numerous physiological, metabolic, and endocrine responses and cues. For instance, WAT regulates appetite, thermogenesis, lipid metabolism, sexual reproduction, immunological responses, insulin signaling, and glucose homeostasis. Moreover, WAT is a highly dynamic organ that can expand and contract depending on the body’s energy demand (7). Because of these attributes and when situated within a positive energy balance—increased food consumption and decreased exercise— it is the perfect recipe for obesity and metabolic dysfunction. However, this is only because evolution, developmental transcriptional programs, and hormonal sex steroids have paved the way for the development of obesity. Indeed, discernable fat-storing tissues can be observed in invertebrates and vertebrates, allowing organismal survival during food restriction or famine periods (8–10). Moreover, these organisms and phyla share conserved developmental transcriptional programs and fat-storing proteins. Specifically, in mammals, sex steroids determine, specify, and expand specific body fat storage depots (11). For example, males tend to accumulate more visceral adiposity, the so-called “apple shape,” which promotes metabolic disorders and increases the risk for cardiometabolic diseases. In contrast, females tend to expand subcutaneous adipose tissue, favoring metabolic protection (**Figure 1**). This review will examine how sex steroids specifically, estrogen, regulate WAT distribution, function, and growth in males and females to control metabolic health.

## Estrogen Action and Signaling Mechanisms

Sex steroids such as estrogens activate a specific group of ligand-dependent transcription factors called nuclear hormone receptors to regulate gene transcription. Estrogens are a group of compounds that include estrone, estradiol, and estriol. Estradiol can be further separated into 17 alpha-estradiol (17α-E2) and 17 beta-estradiol (17β-E2) (12). Specifically, estrogens are synthesized and metabolized by the cytochrome P450 (CYP) superfamily of enzymes (13). Biosynthesized from cholesterol, estrogens are produced using a host of CYP enzymes, the most notable being aromatase (CYP19A1), converting androgens to estrogen (14). Aromatase is found in a variety tissues such as brain, adipose tissue, blood vessels, and bone with highest expression taking place in the gonads (15). Thus, the main site of estrogen biosynthesis in premenopausal females occur in the reproductive track and ovaries (16, 17). In postmenopausal women, WAT becomes the bodies major supplier of estrogen, which is dependent on robust aromatase expression and activity (18). Particularly, in WAT, aromatase converts estrone—a converted metabolite from androstenedione, a secreted hormone from the adrenal gland—to estradiol by the 17-beta hydroxysteroid dehydrogenase (17β-HSD) class of enzymes (19, 20). However, the levels of estrogen produced by this pathway are unable to compensate for the loss of ovarian estrogen production, accordingly hormone replacement therapy (HRT) may be required for post-menopausal women. Nevertheless, 17β-E2 is the major circulating and biologically active form of estrogen. It is also the most described in adipose tissue regulation, and we will use the common term estrogen to refer to 17β-E2, broadly (21, 22).

Estrogen can bind and activate two estrogen receptors (ERs), alpha and beta (ERα; ERβ) (23). Classical ER activation requires the binding of estrogen within the ligand binding pocket of the receptor. Estrogen binding to ER allows the receptor to directly interact with DNA by binding to estrogen response elements of target genes (24). Upon DNA binding, ER can cooperate with other transcription factors and be tethered to transcriptional coregulators to communicate with the DNA polymerase to initiate or repress gene transcription. The ability of ER and estrogen to regulate gene expression is often disrupted in many disease states, such as breast cancer. In these scenarios, control mechanisms no longer regulate estrogen-induced ER transcription, resulting in potential tumorigenic action (25, 26). Moreover, modulating levels of estrogens in circulation also plays a critical transcriptional role in controlling metabolic and disease responses (27). In addition to estrogens, environmental estrogen mimetics such as Bisphenol A (BPA) have been shown to activate or suppress ER activity in various tissues. For example, BPA acts as an ERα agonist but has a significantly lower affinity for ER than estrogen (1000- to 2000-fold less) (28, 29). Chronic exposure to BPA has been implicated in disrupted human health and has been associated with an elevated risk of cancer, developmental malformities, obesity, and infertility (28, 29).

In addition to the traditional activation of ERs, estrogen can also associate with and activate a G-protein coupled membrane-bound estrogen receptor called GPER or GPER30 (30). GPER appears to be critical for driving ER-independent pathways and mediates the non-genomic effects of estrogen (30). Upon GPER activation by 17β-estradiol the MAPK (Erk1/2) and adenylyl cyclase pathways are activated to promote various cellular activities (31, 32). Specifically, GPER has been suggested to promote estrogen-mediated inhibition of oxidative stress-induced apoptosis, increased cellular growth through stimulation of cyclin D expression, and upregulated nerve growth factor production in macrophages (33–36). This unique and diverse biological repertoire has strong implications on breast cancer biology and progression. This range of activity exists for adipose tissue as well, and all ERs and GPER3 are expressed within various WAT depots. Yet, ERα has been the most extensively evaluated and appears to drive most of estrogen WAT-related functions (37, 38). In contrast, the roles of ERβ and GPER are not well characterized; however, both estrogen receptors do appear to regulate metabolism (39, 40).

### Estrogen Cycling in Humans and Rodent

The human menstrual cycle begins at puberty, occurring every 28 days, and consists of three phases, menstrual, proliferative, and secretory (41). However, mice have a more truncated reproductive cycle of five days consisting of 4 phases: proestrus, estrus, metestrus, and diestrus. Furthermore, the reproductive cycle in mice consists of 4 phases, including proestrus, estrus, metestrus, and diestrus, compared to humans. Unlike humans, female rodents do not experience menstruation or menopause, but their reproductive organs do undergo senescence (42). To mirror human menopause, 4-vinyl cyclohexene diepoxide has been used to induce the loss of ovarian follicles in mice. Mice receiving this compound do develop metabolic and cardiovascular disease (43). More commonly, ovariectomy surgeries are performed on mice to mimic human menopause to study the effects of estrogen loss on various tissues such as bone, adipose tissue, and metabolic disease (42).

## The Regulation of Body Fat Distribution by Estrogens

WATs are dispersed and are noncontiguous throughout the body, representing the potential heterogeneity of this organ (44). For example, WAT can be demarcated into two broad anatomical locations, subcutaneous and visceral, which can be further separated into distinct compartments called depots. Subcutaneous fat resides below the dermis while visceral fat surrounds internal organs within the body cavity (4, 6, 8). This gross anatomical placement also appears to have significant metabolic implications. Subcutaneous fat appears to be metabolically protective, whereas visceral fat contributes to metabolic dysregulation (3, 45, 46). This protective anatomical distribution of WAT is particularly relevant between males and females. For instance, women will tend to have 10-20% more body fat than men of the same body mass index (BMI) (47). However, premenopausal females preferentially accumulate subcutaneous fat throughout the lower body, hips, and thighs and have reduced visceral adiposity (48). Moreover, premenopausal women are more protective against developing metabolic disease, likely due to the increased subcutaneous fat to visceral fat ratio. In contrast, males often accumulate excess visceral fat, leading to metabolic disorders and cardiovascular disease (49). However, postmenopausal females often accumulate visceral fat while reducing subcutaneous WAT depots. This effect is predominantly due to the lack of estrogen, predisposing women to metabolic disease (50). Indeed, studies using the data from the National Health and Nutritional Examination Survey (NHANES), the most extensive nutritional assessment data on interview and physical examinations available, showed that an increase in visceral or centralized adiposity is associated with the most significant risk of mortality in women (51–53). A UK study further supported this notion by demonstrating a similar risk of mortality and visceral adiposity in women (51, 52). In general, elevated visceral fat deposition is associated with the highest risks for metabolic disease and premature death, regardless of sex (54). Thus, the overall changes in adipose storage sites and sexual dimorphic responses controlling body fat disposition are thought to explain why men develop cardiometabolic diseases earlier than women.

### The Role of Circulating Estrogens on WAT Location

What might account for these metabolic differences and adiposity between males and females? It seems to emanate from where adipose tissue growth occurs and the bioavailability of estrogens. Accumulating evidence from human and rodent studies has demonstrated that higher estrogen levels augment subcutaneous WAT expansion and blunts visceral WAT growth (37). In addition to body fat distribution, ovarian estrogen levels can further protect against obesogenic cues and metabolic disease (55). Research has shown that lowering circulating levels of estrogen by menopause or ovariectomy increases the risk of developing obesity, type 2 diabetes and cardiovascular disease (56–58). In rodents and humans, estradiol replacement therapy or HRT reverses obesity by lowering visceral fat mass thereby improving metabolic fitness (59, 60). In humans, HRT has been shown to have numerous beneficial metabolic effects in post-menopausal women. For example, in a 3-year study, HRT statistically decreased fasting glucose levels and significantly lowered incidences of diabetes (61). Additionally, HRT elevated HDL while lowering LDL, resulting in healthier lipid profiles (62, 63). Furthermore, postmenopausal women receiving estrogen had a decrease visceral adiposity, which reduced their risk of cardiovascular disease (64). In agreement with these studies, ovariectomized rodents provided with estradiol replacement therapy had decreased food intake and increased energy expenditure, protecting them from fat mass accumulation (65–68). Interestingly, these observations are not confined to women; research suggests that men and male mice also benefit from estrogen activity and signaling. For example, studies have shown that the loss of estrogen signaling in males promotes obesity and impairs glucose metabolism (69–72). Moreover, cross-sex hormonal therapy in trans women receiving estrogen exhibit a more feminine body fat distribution and a lower waist-to-hip ratio (73). Thus, circulating estrogen contributes to body fat distribution, remodeling, and maintenance but how are circulating estrogen levels and ER activity maintained?

### The Role of Estrogen Receptor Alpha on WAT Location

**Figure 2:**
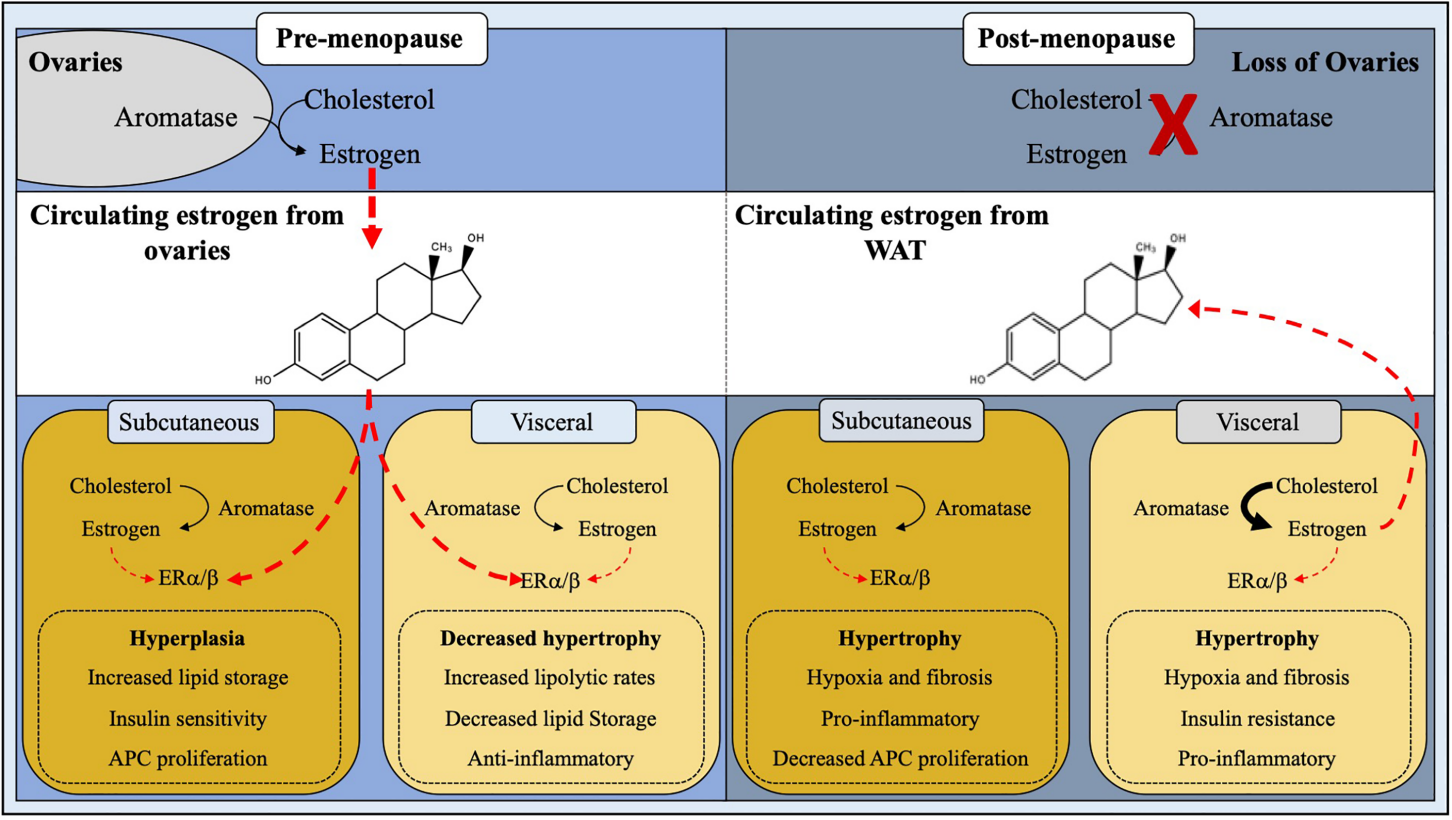
Site-specific regulation of adipose tissue by estrogen. White adipose tissue homeostasis and expansion is influenced by estrogen. In pre-menopausal females, estrogen produced by the ovaries plays a major role in ERα/β signaling thus downregulating androgen receptors, leading to hyperplastic subcutaneous WAT expansion and decreased visceral adipogenesis. Post-menopausal women have a ∼95% reduction in circulating estrogen levels due to cessation of ovarian function, resulting in a stunted ERα/β activation, thus giving rise to metabolically unhealthy hypertrophic WAT expansion.

Modulating estrogen impacts adiposity in pre-and postmenopausal women but is this effect driven by ERs? In overweight to obese premenopausal women, ER expression differences can be observed in abdominal and gluteal adipose tissues (74). These data suggest that receptor availability may be critical for thwarting regional fat depots in response to a positive energy balance (**Figure 2**). A human study comparing regional differences in abdominal and femoral subcutaneous in pre-and postmenopausal women, found that metabolic differences were ER isotype expression dependent (75). That is, these metabolic differences (i.e., insulin sensitivity) emanated from the alterations in the ERα and ERβ ratio between the two fat depots. A higher ERα to ERβ ratio was observed in premenopausal women than in postmenopausal women (75). Moreover, treating both pre and postmenopausal subjects with 17β-estradiol increased the ERα to ERβ ratio within WAT, suggesting that estrogens mediated changes in the ER ratio is critical for inducing insulin sensitivity of WAT (75). While interesting and physiologically relevant, the mechanisms governing this response remain to be elucidated. A potential regulatory mechanism accounting for these changes in the ERα:ERβ ratio may be due to enhanced ERα promoter silencing *via* DNA methylation. This appears to occur in the rat aorta, but studies examining ERα promoter methylation in fat are lacking and cannot be linked to metabolic disease (76). What is clear is the consistent increase in adiposity, specifically visceral WAT, and altered energy metabolism of ERα null male and female mice (38). This obesogenic and altered energy balance in ERα null mice is further heightened when fed a HFD (77).

Yet, the increase in adiposity in ERα null mice might be independent of WAT biology and may be linked to hypothalamic estrogen-ER regulation. For example, knockdown of ERα within the ventral medial nucleus (VMN) caused weight gain *via* disruption in energy expenditure independent of food intake. Additionally, knockdown of ERα within the VMN results in metabolic dysfunction in rats. Yet, estrogen-induced effects on food intake can be observed in response to low-dose microinjections of 17β-estradiol into the brain. In agreement, reports have suggested that estrogen decreases orexigenic peptides to reduce food intake. For example, a reduction in neuropeptide Y (NPY), a potent appetite stimulator, has been associated with increased circulating estrogen levels and estrogen-mediated ER activity (78, 79). Specifically, NPY transcription corresponds to the ERα:ERβ ratio present within the hypothalamus. When the ratio is high, there is less NPY transcribed; conversely, when the ratio is low, NPY is produced (80). Additionally, changes in the level of the hunger hormone ghrelin have been associated with different phases of the ovarian cycle (81). Specifically, ghrelin infusion during diestrus one and diestrus two stimulates eating but not during proestrus or estrus, coinciding with the peak in estrogen levels. In support of this notion, ovariectomized ghrelin receptor knockout mice do not develop hyperphagia or body weight gain. This suggests that reducing circulating levels of estrogen increases food intake by releasing ghrelin from a tonic inhibitory effect of estrogen. Thus, estrogen normally suppresses ghrelin function to block food intake, but in the absence of estrogen, ghrelin secretion is unchecked, mediating the hyperphagic response (81). It remains unclear how estrogen affects ghrelin-mediated eating and whether these effects can originate from ghrelin signaling, secretion, or receptor expression.

### The Role of Estrogen Receptor Beta on WAT Location

While ERα gene ablation leads to a distinct and robust metabolic dysfunctional response, ERβ gene deletion appears less obvious. Interestingly, the whole-body deletion of ERβ has relatively no effect on adiposity or energy balance (82). This observation led to the hypothesis that ERβ promoted obesity and metabolic disorders. This observation was further supported by a 10-fold increase in 17β-estradiol concentration in ERα null mice, thus, inferring an increased flux in estrogen signaling through ERβ (83). In support of this view, ovariectomized ERβ null mice were protected from obesity (40). However, follow-up studies showed that ERβ null mice were actually more susceptible to obesity but protected against insulin resistance (84). This obesogenic effect on ERβ null mice was further heightened by ovariectomy. In alignment with these rodent studies, a human genetic study revealed five single nucleotide polymorphisms (SNPs) in ERβ associated with obesity in both males and females (85). Yet, the molecular mechanisms and regulatory features of ERβ under diet-induced obesity remain to be elucidated. But new studies are beginning to suggest that ERβ may have a metabolic protective role by regulating WAT mitochondria activity. For example, in rodents, ERβ specific ligands appear to increase energy expenditure and WAT mitochondria activity, even in the absence of circulating estrogens (86, 87). Moreover, changes in fat storage genes and adipokines have been related to the activation of ERβ over ERα or the ratio between the two receptors (88, 89). In totality, these studies would argue that both ERα and ERβ are required for proper energetics to mediate metabolic flexibility. Collectively, the area of ER isotype activation, ER expression profiling, ER receptor-DNA interaction, and non-genomic activities of estrogen are critical areas of investigation and will help resolve unanswered questions.

### The Role of GPER Activity on WAT Location

In addition to ligand-receptor genomic mediated responses, estrogen can also have non-genomic responses mediated by GPER (G-protein coupled membrane-bound estrogen receptor). GPER is expressed in intracellular membranes, and like other GPCRs, it couples estrogen signaling to changes in adenylyl cyclase, kinase, and ion channel activity. Notably, GPER can also indirectly regulate target gene expression. GPER is expressed in a host of tissues; however, GPER does not appear to be involved in estrogen-mediated reproduction (90). This is because GPER null mice are fertile, whereas ERα null mice are infertile (91). Within adipose tissue, the metabolic roles of GPER remain elusive, but in recent years more information has been garnered, which has been recently reviewed in (92). Briefly, some studies show that GPER deficiency leads to reduced body weight and bone growth in females, and other studies reported a significant increase in fat mass when GPER is deleted (93–96). In contrast, several studies have shown an effect of GPER or its activation on body weight even in response to HFD (97, 98). Overall, the ability of estrogen to regulate fat mass and sexual dimorphic distribution relies on receptor expression, estrogen bioavailable and synthesis, and age.

### The Role of Aromatase on WAT Location

What might control local and systemic estrogen levels to regulate body fat distribution? The answer to this question appears multifaceted but may be related to WAT CYP19 gene (aromatase)—the enzyme required to synthesize endogenous estrogen—expression and activity (**Figure 2**). In agreement, knockout mice lacking the functional aromatase gene are markedly obese (99). In contrast, activating aromatase within WATs reduces adiposity and improves metabolic performance, such as insulin sensitivity (100). Notably, only male mice with heightened aromatase activity showed changes in body fat distribution. This effect could be attributed to overall changes in adipose tissue estrogen levels in males but not females, presumably due to the already elevated amounts from ovarian estrogen (100). Genetic necessity and sufficiency tests have demonstrated the importance of aromatase enzyme in estrogen bioavailability and WAT function, but there are additional aromatase regulation layers. For example, the human aromatase promoter contains eight unique start sites allowing for selective tissue expression (101, 102). Because aromatase has a diverse tissue expression profile, it could be inferred that the additional regulatory mechanisms, such as epigenetic DNA regulation, exist to govern tissue specificity and spatiotemporal control. Regarding epigenetic regulation, the aromatase promoter has been shown to be highly methylated, which appears to be evolutionarily conserved among reptiles, fish, birds, ungulates, and primates (103–107). Yet the mechanisms driving DNA methylation of the aromatase promoter remains obscure and substantially less is known for WAT. A potential modulator of aromatase epigenetic modification may be driven by microRNAs (miRNA; miR). miRNAs are small 20-30 nucleotide long regulatory RNA molecules that have implications in numerous biological processes, including WAT physiology (108). For example, we have previously shown that the conserved family of miR-26 miRNAs can regulate adipogenesis and WAT mass by repressing Fbxl19, an E3 ubiquitin ligase complex (109). Consistent with this notion, miRNA activity appears to be linked to aromatase gene suppression in human breast cancer. However, the full spectrum of miRNA and DNA methylation regulation remains undefined for WAT, which could have implications on visceral and subcutaneous expansion. In agreement, a recent study by Martinelli et al., compared obese and lean WAT from males and females and identified 42 differentially expressed miRNAs (110). Yet, the molecular roles of these various miRNAs are unclear but suggest that miRNAs could be an essential genetic regulatory feature controlling estrogen levels and adiposity.

Aromatase expression can also be induced by cAMP activators such as protein kinase A and C (111). Indeed, inhibition of phosphodiesterase type 5 (PDE5), an enzyme that breaks down cAMP and cGMP, increases aromatase gene expression within cultured human visceral adipocytes (112). The upregulation in aromatase expression by PDE5 inhibition boosts estrogen production, which could be protective against metabolic dysregulation. While the clarification of the pathway remains controversial, it may depend on the identification of two distinct cAMP binding protein (CREB) response elements within the aromatase promoter (113). Regardless of modality, inhibition of PDE5 and its subsequent increase in visceral WAT estrogen production could induce anti-inflammatory responses and improve vasodilation (114). Yet, it remains to be determined if PDE5 inhibitors can improve systemic metabolism and counteract visceral WAT expansion, especially under estrogen depletion methods (ovariectomy and aromatase knockout mice) (115).

## Storing and Expanding Fat

WATs expand and contract in response to metabolic demands and overnutrition. Accordingly, WAT can expand *via* two main methods: hypertrophy and hyperplasia (116, 117). Adipocyte hypertrophy is characterized by lipid filling of existing adipocytes to store excess nutrients as triglycerides. Studies in rodents and humans have identified that WAT expansion by adipocyte hypertrophy is considered metabolically unhealthy. Presumably, this is because as adipocytes swell to accommodate extra storage, their transcriptional program, adipokine secretion profile, insulin responses, and lipid metabolism and capacity become disrupted (4, 116, 117). Yet, modulation of adipocyte size regulators, such as SWELL1, are required for proper adipocyte lipid function and glucose sensing (118). Moreover, a hypertrophic response is associated with chronic low-grade inflammation, immune cell composition alterations, and fibrotic tissue replacement (119) (**Figure 1**). Subsequently, changes in immune cell populations stimulate pro-inflammatory signals to recruit more immune cells to facilitate adipocyte cell death. Adipocyte death is accompanied by the release of triglycerides into circulation, which, over time, will cause hyperlipidemia and ectopic lipid accumulation. In contrast to hypertrophy, WAT hyperplasia is associated with APC proliferation and expansion with subsequent adipogenesis (120). New smaller and potentially healthier adipocytes positively affect WAT health protecting against hyperlipidemia and insulin resistance. Additionally, WAT hyperplasia promotes metabolically favorable immune cells, promoting an anti-inflammatory environment, reducing inflammation and adipocyte cell death (120). Likewise, hyperplasia is associated with WAT angiogenesis, providing the tissue with oxygen, nutrients, and the ability to enlarge—as it serves as a platform for APC growth and viability (121). Thus, WAT hyperplasia—while it does lead to an increase in adipose tissue mass—appears to be a protective measure against the rapid onset of metabolic dysfunction (**Figure 1**).

Adipose tissue mass balances adipocyte number and volume, which is tightly regulated and appears to depend upon various dietary stimuli and nutrient availability (excess or deficiency). However, *in vivo* adipocyte kinetics (turnover and expansion) also appears to be controlled by the presence and abundance of sex steroids. For example, in women, adipocyte diameter tends to be greater in subcutaneous WAT than visceral WAT (122). On the other hand, adipocyte size appears to be equivalent in males and obese females’ fat pads (123). Studies in rodent models have demonstrated sex-dependent differences in WAT depot remodeling by hypertrophy versus hyperplasia. In response to a caloric excess, male gonadal fat pads mainly expand through hypertrophy, whereas female visceral and subcutaneous adipose tissue expands *via* both methods (124). Consequently, there is more adipocyte cell death and inflammation in male fat pads than in female WAT (125). Moreover, high-fat diet (HFD) fed female rats have a delayed onset of insulin resistance and type 2 diabetes compared to male littermates (126). Indeed, the usage of the ERα null mouse has revealed preferential expansion of WAT depots, specifically visceral, that foster metabolic imbalance (38). However, an essential caveat of the whole-body ERα gene deletion is a disruption in energy balance from non-adipose tissues, such as hypothalamic regulation on energy homeostasis (127). Yet, recent advances by Clegg and colleagues have demonstrated that a novel visceral depot-specific ERα-siRNA knockdown strategy showed a reduction in ERα expression increased visceral WAT weight and adipocyte size (67). These data tend to support the direct effects of ERα on adipocyte biology. The following sections will highlight various areas of estrogen action on WAT hypertrophy and changes in metabolic consequences (**Figure 2**).

### The Role of Estrogens in WAT Lipid Metabolism

The regulation of adipocyte size directly links to triglyceride storage and lipolysis. Lipid storage is influenced by the rate of fatty acid uptake and its conversion into triglycerides. On the other hand, depending on metabolic demand, triglycerides can be hydrolyzed and released into circulation to fuel target tissues. This balance between storage and breakdown, in part, can be ascribed to the bioavailability of sex steroids. It has been reported that the majority of circulating fatty acids in women are taken up by subcutaneous fat (128). In contrast, a significant portion of dietary fats are preferentially stored in male visceral fat (129). This favored partitioning of fatty acid uptake in male visceral fat may not be due to inherent sex steroid differences. Instead, it may be related to extracting lipids from chylomicrons due to the proximity of visceral fat to the digestive track (130). Yet, estrogen appears to have a direct role in suppressing lipid storage genes and preferentially activating lipolytic pathways. For instance, in adipocytes and cancer cells, ER receptor has been shown to directly interact with peroxisome proliferator activated receptor gamma (Pparγ), a major driver of lipid storage and adipogenesis, to block its transcriptional activity (131, 132). Blocking Pparγ activity has several implications in both the adipose progenitor and adipocyte compartments. For instance, estrogen-mediated blockade of Pparγ activity in APCs would prevent adipogenesis and hinder the potential metabolic benefit of hyperplasia (133). In contrast, suppression of Pparγ transcriptional activity within mature adipocytes by estrogen would repress lipid biogenesis and insulin sensitization genes (134). As noted above, these effects of estrogen on Pparγ may be depot specific and may have additional transcriptional regulatory steps controlling Pparg target genes. In addition to Pparγ, estrogen and estrogen levels have been shown to directly repress lipoprotein lipase (LPL) gene transcription and decrease LPL activity through posttranscriptional modifications (135). For example, clinical analyses of serum triglyceride levels are increased in postmenopausal women but are normalized or even reduced in response to HRT (136). Similarly, in obese women, lower fasting LPL activity was associated with higher levels of circulating estrogen (137). Critically, LPL activity is responsible for the conversion of triglycerides into free fatty acids, allowing free fatty acid uptake into non-hepatic tissues (138). Thus, reducing LPL expression or decreasing its activity through posttranscriptional modifications will dampen free fatty acid uptake. However, it is unclear if estrogens regulate LPL activity and gene expression in a WAT depot specific manner. In general, largescale genetic transcriptional studies examining the role of estrogen signaling in regulating subcutaneous and visceral adipocyte gene expression appear to be lacking. Studies focused on understanding sex-steroid and gene interplay could help resolve these associations and observational studies to describe differences in male and female WAT adipose tissue distribution and activity.

Estrogen may also facilitate adipocyte lipolytic rates. Estrogen activation of ERα appears to upregulate the antilipolytic alpha2A-adrenergic receptors only in subcutaneous WAT but not visceral (139). In addition, examination of fat distribution in women suffering from polycystic ovarian syndrome (PCOS), a disease state in which the ovaries overproduce androgens, has shown a preferential expansion of visceral WAT with a reduction in subcutaneous WAT (140). These changes in adipose storage and remodeling in PCOS patients are believed to result from plasma androgen levels (141, 142), which blocks lipolysis and stimulate lipogenesis in the visceral compartments (40). Moreover, estradiol-treated ovariectomized mice showed enhanced lipolytic responses, favoring free fatty acid oxidation and not storage. This lipolytic effect could be attributed to estrogen-induced gene expression changes in the fatty acid oxidation nuclear hormone receptor, Pparδ, and its fatty acid oxidation pathways (142, 143). Also, lipid oxidation may require estradiol’s non-genomic activity to activate AMP-activated protein kinase, rapidly (142). Yet, more information is needed to understand the full spectrum of estrogen’s effects on lipolysis, lipolytic rates, and gene expression of lipolytic and lipogenic genes.

### The Role of Estrogens in Regulating WAT Adipokines

Beyond the gross anatomical placement of adipose tissue, estrogens also appear to regulate WAT endocrine function. Notably, changes in adipocyte endocrine function in response to hypertrophy have considerable effects on appetite, glucose metabolism, lipid uptake, thermogenesis, and reproduction status. Adipocytes regulate physiology and systemic metabolism by secreting signaling molecules called adipokines. Adipokines can have paracrine, autocrine, and endocrine functions, but become disrupted in response to chronic overnutrition. For example, leptin is an adipokine synthesized and secreted into circulation from adipocytes, which can regulate energy balance and appetite suppression through hypothalamic neurons (144, 145). Leptin expression levels in WAT and amounts in circulation are tightly correlated with fat mass. Obese individuals tend to have elevated circulating levels of leptin and upregulation of leptin gene expression in WAT; however, these individuals appear to be unresponsive to leptin action. Indeed, obese patients tend to be leptin resistant and no longer respond to leptin-induced anorexigenic suppressing signals. In agreement, patients with leptin resistance and rodent models lacking leptin or the leptin receptor are hyperphagic (145). The mechanisms underlying human leptin regulation and resistance are not yet fully elucidated, but sex steroids such as estrogen may be an entry point. Consistent with the notion of elevated fat mass and leptin, ERα knockout mice, which have superfluous adiposity, also have more leptin in WAT and circulation. But these animals may be leptin resistant because they are hyperphagic (38). There are also correlations between sex steroid abundance and leptin gene expression and circulating levels. For instance, women tend to have higher serum leptin levels than men (146). This elevation in leptin can even be observed *in utero* and persist throughout sexual maturity and life (147). The differences in leptin levels between males and females may not be attributed to changes in fat mass but rather through direct regulation. For instance, testosterone is negatively correlated with leptin levels, whereas estradiol positively regulates leptin levels (148, 149). Interestingly, postmenopausal women—who have higher visceral adiposity—tend to have less circulating leptin, which corresponds to less leptin gene expression within WATs (150). Cell culture studies on *in vitro* derived adipocytes from female patients suggest that estrogen increases leptin production and secretion whereas male derived adipocytes do not (151). In contrast, treating human adipocytes with testosterone decreases leptin levels and secretion (148). Similarly, treating aromatase null mice, which are also obese and have elevated serum leptin levels, with estradiol can restore leptin to wild-type levels (152). Moreover, it appears that estrogen may directly regulate hypothalamic action to regulate eating behavior by reducing endoplasmic reticulum stress within the ventromedial nucleus, enhancing the sensitivity to leptin (145). This effect may also be, in part, due to reducing ceramide levels within the hypothalamus (153). Yet, why women have higher levels of leptin remains unidentified.

In addition to leptin, estrogens have been shown to regulate another WAT specific adipokine, adiponectin (154). Metabolically, adiponectin has been shown to improve insulin sensitivity by increasing pancreatic insulin gene expression and enhancing its secretion (155). Moreover, adiponectin suppresses hepatic glucose production, promotes anti-inflammatory signals, and enhances fatty acid oxidation in the liver and skeletal muscle (154). Interestingly, adiponectin levels are lower in aged and BMI-matched males than females (156). During puberty, as androgen levels surge, sexual dimorphic changes in circulating adiponectin levels can be observed (157). In agreement, surgical castration increases adiponectin levels, whereas testosterone supplementation lowers circulating adiponectin levels (156). Yet modulating estrogen levels do not appear to be straightforward. For example, ovariectomized rats do not demonstrate changes in adiponectin levels even though visceral WAT is augmented. In addition, treating ovariectomized rats with estrogen reduced fat mass and improved metabolic health but did not elevate adiponectin levels (158). Yet, Scherer and colleagues demonstrated that ovariectomized adult female mice had elevated adiponectin levels (159). Moreover, it was shown that estrogen treatment suppressed adiponectin in mice and *in vitro* derived adipocytes. Interestingly, neonatal castration allowed adiponectin levels to reach female adult levels (159). Thus, changes in circulating levels of adiponectin between males and females may be predominantly driven by male sex steroids. This is further suggested by changes in adiponectin circulating oligomeric complexes. Adiponectin circulates in low, medium, and high molecular weight oligomeric forms in serum (154). The ratio of high molecular weight forms of total adiponectin may be critical for its ability to regulate insulin sensitization and cardiovascular function. Changes in adiponectin levels between males and females have also been associated with changes in high molecular weight adiponectin forms (160). Yet, these changes also appear to be governed by androgens, as testosterone has been shown to reduce higher molecular weight forms of adiponectin (156). Specific reductions in adiponectin oligomeric states could be a potential mechanism for higher cardiometabolic diseases in males than females.

### The Role of Estrogens in WAT Immunological Function

Chronic low-grade inflammation of WAT is a significant rheostat of adipocyte function and health. Under homeostatic conditions, WAT is predominantly associated with M2 macrophages that maintain tissue health by promoting anti-inflammatory signals. However, chronic overnutrition forces immunological changes that favor M1 macrophages to perpetuate pro-inflammatory cytokine signaling to trigger adipocyte cell death (161). For example, a dysfunctional adipocyte can recruit M1 macrophages to WATs by secreting pro-inflammatory adipokines such as interleukin 6 (IL-6) and tumor necrosis factor-alpha (TNFα). Recruited M1 macrophages form a crown-like structure, surrounding the dysfunctional adipocyte then engulfing it. Stored lipids are then released into circulation for potential ectopic lipid storage (162). Visceral WAT appears to be more susceptible to obesity-driven inflammatory signals and is the main culprit in producing and secreting IL-6 and TNFα than subcutaneous fat (163). Changes in visceral WAT cytokine and pro-inflammatory, secretion, and M1 macrophages recruitment and activation, appear to be a vicious cycle, putting the tissue into a “hyperinflammatory” state (164, 165). This heightened state of inflammation significantly fosters and bolsters insulin resistance, hypertriglyceridemia, and continued chronic low-grade inflammatory responses (161). WAT inflammation is thought to be one of the major drivers of metabolic dysfunction and ensuing WAT dysregulation.

Tissue-specific innate immune responses through pro- and anti-inflammatory processes appear to be regulated by estrogen availability. A key determinate of estrogen action is bioavailability and concentration, immune cell type, immune stimulus, and receptor expression (166). Yet broadly, it appears that estrogen may suppress pro-inflammatory signals. For instance, menopause or surgical menopause, oophorectomy, increases the number of proinflammatory markers but can be alleviated by estrogen replacement therapy (167). In agreement, estrogen-treated ovariectomized mice have increased M2 macrophages accompanied by elevated anti-inflammatory markers (168). For WAT, estrogen appears to suppress pro-inflammatory responses and promotes anti-inflammatory signals. In agreement, mouse models of either adipocyte or macrophage ERα deletion showed increased WAT inflammation (67, 169). These results may emanate from expression patterns of ERs within macrophages. It appears that macrophages express higher levels of ERα compared to ERβ (170). In alignment with this notion, macrophages lacking ERα have a significantly higher lipopolysaccharide-induced TNFα release (169). Specific myeloid deletion of ERα showed altered adipokine and cytokine levels, glucose intolerance, insulin resistance, and increased WAT mass. Additionally, in isolated macrophages, ERα appears to be critical for interleukin-4 mediated alternative macrophage induction, favoring metabolic and anti-inflammatory protection. Further, loss of macrophage ERα expression accelerated atherosclerotic plaque development in female mice (169). Consistent with estrogen-mediated inflammatory responses, whole-body aromatase gene ablation resulted in an upregulation in TNFα and IL-6 and the recruitment of the pro-inflammatory M1 macrophages (99). On the other hand, increasing WAT estrogen biosynthesis *via* aromatase overexpression reduced WAT inflammation (82). Likewise, treating male mice with estradiol reduced macrophage and inflammatory markers, which was linked to improved insulin sensitivity (100). *In vitro* modeling using isolated macrophages and adipocytes has shown similar results; that is, estrogen treatments can effectively reduce IL-6 and TNFα levels (67, 171, 172). Interestingly, ERβ null female mice appear to be protected against inflammation and fibrosis, possibly through enhanced estrogen-ERα signaling within macrophages (40, 82). Moreover, the ER-dependent repressive pro-inflammatory mechanisms within macrophages appear to be driven by modulating cytokines and genes involved in activating the nuclear factor kappa b (NFκB) pathway. These effects on NFκB signaling appear to more well developed in cancer tumor-associated macrophages (173). Overall, estrogen and ER activity seem to influence WAT inflammation; however, these effects may be attributed to changes in macrophages and adipocytes. Further studies at elucidating specific roles of ER and estrogen in adipocytes and immunological cell types will provide direct mechanistic and transcriptional evidence for estrogen regulation.

### The Role of Estrogens in WAT Vascularization

For proper adipose tissue health and growth, vascular expansion by angiogenesis is required (174). As with most tissues, an appropriate supply of oxygen and nutrient delivery and removal requires the blood vessel system. Moreover, hormones and growth factors fluctuating into and out of the tissue to support tissue function, homeostasis, and cellular respiration utilize the vasculature system as a conduit for transport. But unlike most organ systems within the body, adipose tissue is highly plastic and can expand from 4% to 50-70% of an individual’s body composition (44). Thus, the requirement of angiogenic potential for WAT is considerably high to facilitate such flexibility. Moreover, the WAT vasculature also serves as a scaffolding for APCs to reside and interact with to promote adipogenesis and facilitate angiogenic action (121, 133, 175). However, upon obesogenic signals, the angiogenic processes within WAT go awry. Increased caloric consumption can stimulate WAT growth by hypertrophy, and this demand for WAT expansion creates “pockets” of hypoxia. Under this state, hypoxic signals and other vascular stimulating factors are released to promote angiogenesis by upregulating the expression and secretion of vascular endothelial growth factor A (VEGFA) (174, 176). However, this process becomes disrupted in obese WAT for reasons not entirely understood. What is clear is the notion that VEGFA can protect against diet-induced obesity (177, 178). For example, overexpression of VEGFA within adipocytes or the adipose lineage promotes WAT vascularization to suppress lipid accumulation, inflammation, and HFD-induced insulin resistance (133, 178). Further complicating this notion is the observation that subcutaneous and visceral fat depots differ in their amount of vascularization (179). The changes in vascularity in various depots could represent variations in nutrient availability, oxygen diffusion, APC number and interaction, and metabolites. Specifically, when considering visceral WAT, blood flow and proximity to metabolically active organs such as the liver and intestine could influence visceral WAT depot expansion and susceptibility to metabolic dysregulation. Moreover, this anatomical visceral WAT proximity further increases its exposure to triglycerides, cholesterols, and glucose (180).

Because estrogen can regulate subcutaneous growth and block visceral WAT expansion, researchers have examined if estrogen modulates the angiogenic potential of various WAT depots. Indeed, postmenopausal women tend to have a reduction in adipose tissue blood flow, increasing their susceptibility to metabolic disease. The data also suggest that ER activation positively regulates VEGFA gene expression (181). In agreement, blocking ER activity showed decreased VEGFA gene expression, resulting in adipocyte hypertrophy, inflammation, and insulin resistance (182). Estrogen signaling has also been shown to regulate angiotensinogen in the liver. Angiotensinogen has been shown to regulate blood flow and pressure and is often elevated in obese patients, augmenting obesity-induced hypertension. Consistent with this notion, angiotensinogen is expressed and secreted by adipocytes and becomes elevated within hypertrophic adipocytes (183). Additionally, in mouse models, it appears that adipocytes are the major source of angiotensinogen and can account for changes in systolic blood pressure in response to HFD (184). Yet, it is unclear if estrogen is a mediator of angiotensinogen expression and activity (185). It is well documented that estrogen can regulate blood vessel biology in the context of atherosclerosis and hypertension and that postmenopausal women lose protection from these conditions. For example, perivascular adipose tissue regulates vascular tone, but in postmenopausal women, perivascular WAT expands, augmenting arterial dissection (186). While these clues highlight that estrogen regulates vascular biology and tone in WAT and other organ systems, it appears that the molecular and transcriptional mechanisms remain to be fully determined.

### The Role of Estrogens in WAT Fibrosis

In response to overnutrition, uncontrolled WAT expansion and accumulation can also trigger fibrosis. Typically, fibrosis is described as the scaring of tissue after injury, occurring through the development and accumulation of fibrous connective tissue or nodes (187). While this process occurs under the normal healing process, fibrosis is also associated with several pathologies such as obesity and liver cirrhosis that promote damaging effects on tissue function, integrity, and homeostasis (188). Nevertheless, adipocytes are normally surrounded by extracellular matrixes that function as a mechanical support lattice and allow WATs to be highly flexible to the body’s energy demands (189). But like with other fibrotic diseases, fibrotic WAT is a response to the accumulation and overproduction of extracellular matrix proteins, creating a thickening web of collagen intertwining between adipocytes and encasing the WAT depot (119). Once initiated, WAT fibrosis can accelerate changes in tissue inflammation, stiffening, and adipocyte cell death. However, like other aspects of obesity, not all patients will develop WAT fibrosis. For that matter, it is not completely understood how chronic overnutrition recruits or stimulates fibrotic signals or the exact mechanisms sustaining a fibrotic response. Sex steroids have also been implicated in the development of fibrosis, but this appears to be complex and heterogeneous and varies among tissue types. For example, in systemic sclerosis, several studies have shown that estrogens induce fibroblast dysfunction to stimulate the production and deposition of extracellular matrix proteins (190). In contrast, other studies have demonstrated that estrogen therapy reduces connective tissue buildup (191). In adipose tissue, the deletion of ERα increases fibrotic tissue formation along with inflammation (67). But there was no improvement in ERα knockout mice WAT fibrotic gene expression in response to ovariectomy (82). While the data are limited, there may be an association between the ability of estrogens to regulate vascularity and fibrosis. As noted above, hypertrophy is often associated with diminished vascularization and tissue oxygenation, which are thought to be the main drivers of fibrosis. However, more research aimed at disentangling the effects of estrogen on vascularity and fibrosis will be needed to understand the molecular and cellular mechanisms.

## A Progenitor Perspective

### Deciphering a Convoluted Lineage

**Figure 3:**
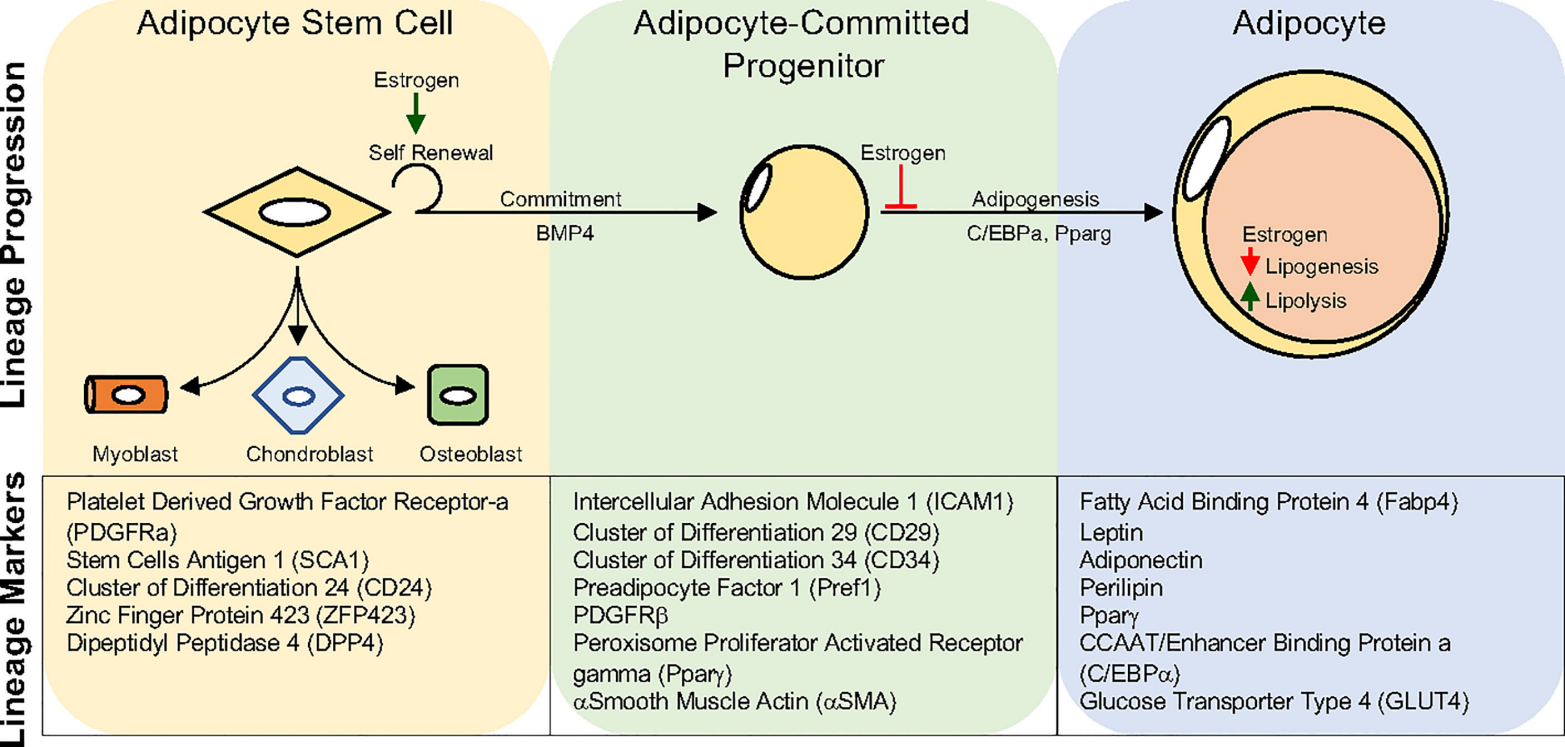
Adipocyte Lineage progression. Adipocyte stem cells can generate committed progenitors which can undergo adipogenesis to become lipid-laden adipocytes. Developmental fate mapping, flow cytometry, and single-cell sequencing methodologies have identified unique and overlapping markers for each step during lineage maturation. Estrogen has been shown to regulate various aspects of the adipocyte lineage progression and mature adipocyte function. Yet, estrogens role may vary depending on depot and sex.

Like many organ systems, adipocytes appear to have a stem/progenitor cell pool capable of undergoing adipogenesis to create new adipocytes. While the transcriptional mechanisms governing adipogenesis have been elegantly and extensively elucidated *in vitro*, our understanding of *in vivo* adipocyte differentiation has languished (182). Not until the mid-2000s did prospective flow cytometric analysis and development lineage tracing techniques become employed to identify potential APCs (192). For example, Friedman and colleagues utilized flow cytometry of potential stem cell markers to identify a Cd24+ cell population capable of making adipocytes *in vitro* and when transplanted into lipodystrophic mice (192). Extending these findings, Berry and Rodeheffer showed that APCs expressing Cd24 could become Cd24 negative APCs, representing a preadipocyte (193). Concurrently, Graff and colleagues harnessed developmental fate mapping tools to identify that APCs express Pparγ (121). In addition to expressing Pparγ, these APCs also expressed a host of smooth muscle genes. Indeed, follow-up studies demonstrated that mural cell genetic tools could mark APCs that had adipogenic potential *in vitro* and *in vivo* (133, 194, 195). These pioneering discoveries led to an explosion of developmental and fate mapping/lineage tracing tools to understand APCs and define their developmental and cellular origins (193, 196, 197). Recently, using the resolving power of single-cell technologies to examine the adipose lineage, it has been identified that APCs express *bona fide* stem cell markers such as Cd24, Cd29, Cd34, stem cell antigen-and 1 (Sca1)/LY6A (198–202). Beyond classical stem cell markers, APCs can be further specified because they express platelet derived growth factor receptor alpha (Pdgfrα), Pdgfrβ, zinc finger protein 423 (Zfp423), and Pparγ (121, 203–207). The identification of white APCs provides a new opportunity to understand if new adipogenesis can facilitate tissue health and growth in response to a positive energy balance. Moreover, researchers can now directly investigate if and how sex steroids control transcriptional programs within APCs to contribute to fat cell development and maintenance (**Figure 3**).

### Tamoxifen-Inducible Mouse Models to Study WAT Biology

To investigate adipose lineage dynamics and APC function, researchers are increasingly using inducible CRE genetic mouse models that require the synthetic partial ER agonist and antagonist, tamoxifen (TMX), for activation. Broadly, CRE-loxP systems offer powerful resolution on lineage analysis, cellular fate mapping, and gene necessity and sufficiency tests in animal models (208). Traditional straight CRE genetic strategies, while helpful, have significant caveats when assessing gene function and lineage analysis on tissue development and homeostasis (209). This is mainly because of the inability to selectively modulate the CRE-driver expression and activity within a specific cell type at a particular time. TMX-inducible CRE systems bypass these caveats to target genes within specific cell populations at precise developmental and adult time points. To provide this type of precision, inducible CRE mouse models contain an estrogen ligand-binding domain fused to a CRE recombinase, CRE-^ER^ (210). Subsequently, these Cre-^ER^s have been modified to allow specific binding of TMX (Cre^ERT2^) and to reduce endogenous estrogen binding (211). Nonetheless, because TMX is a biologically active compound, several effects on WAT biology have been reported. For example, the administration of TMX can influence the appearance of beige adipose tissue within WAT. Yet, these effects of TMX may also be dependent on the mode of administration, for instance, intraperitoneal (IP) injection versus oral gavage. Typically, TMX is metabolized in mammalian systems in five to seven days, but within WAT, TMX metabolism may be delayed (~15 days) (212). Further, it was reported that CRE expression remains within the nucleus after TMX, yet it is unclear if this is related to CRE expression or activity? What is lacking regarding TMX inducible systems is the optimization of TMX dosage and duration for each unique driver and reporter combination. Typically, researchers use a TMX dose of 100 mg/Kg administered for five days by IP (197, 213). But this may be unnecessary. For example, flow cytometric analysis of WAT stromal vascular cells from the alpha-Smooth muscle actin (Sma)-Cre^ERT2^ mouse model combined with the indelible reporter model, Rosa26^tdTomato^, showed a 75% recombination efficiency (Sma-RFP+/total Sma+ cells) after two days of 100 mg/Kg IP TMX administration (195). This equates to 2.5x less tamoxifen than typically used. Therefore, careful examination of recombination efficiency and dosage should be considered when using TMX-inducible genetic models to avoid untoward side effects.

### The Role of Estrogen in Regulating WAT Development

But how might sex steroids regulate APC biology during WAT organogenesis and homeostasis? In humans, adipocytes are specified and lipid-filled during gestation, whereas in rodents, adipocyte lipid filling occurs postnatally (195, 214, 215). Critically, in humans, fetal fat development directly affects childhood fat mass accumulation and their susceptibility to obesity (216). The developmental origins of white adipocytes have been suggested to emanate from a mesodermal cellular source; however, this notion had never been rigorously tested. Recent findings by Sabo and Rodeheffer provided direct genetic fate-mapping evidence to show that subcutaneous and visceral adipocytes originate from a mesodermal origin (217). Interestingly, not all fat derives from mesodermal cells. Using chick-embryo chimeras, elegant studies from Billon and colleagues, identified that adipocytes surrounding salivary glands develop from a neural crest cellular origin (218). Adding further to the fray was the identification that distinct mesodermal progenitors develop visceral WAT but not subcutaneous adipocytes. Hastie and colleagues demonstrated that Wilms Tumor 1 (Wt1) expressing cells within the mesothelium generate only visceral adipocytes (219). Yet, it is not entirely understood how sex steroids regulate WAT development, adipose lineage specification, and WAT depot patterning. As early as puberty, changes in body fat mass and distribution can be observed in humans, suggesting that sex steroids can influence WAT organogenesis and homeostasis (54). Still, again, the actions mediating WAT development by sex steroids remain unknown. Additionally, sex steroids may not be the only driver of fat remodeling; Chen et al. showed that the number of X and Y chromosomes affect adiposity and metabolism, independent of sex-steroid levels (220). For example, mice with a lone X chromosome have a growth deficiency compared to XX and XY mice due to haploinsufficiency (221, 222). In humans, women with a lone X chromosome, known as Turner syndrome, have impaired hormone levels resulting in a 4-fold increased risk of developing type 2 diabetes and metabolic syndrome (223). Another example is Klinefelter syndrome (XXY), a common sex chromosome disorder resulting in hypogonadism in males, in which patients will have a five-fold increased risk of developing abdominal obesity, elevated fasting blood glucose levels and triglyceride levels, reduced HDL, and hypertension (224, 225). Additionally, they have an increased risk of developing type 2 diabetes (226). Collectively, WAT development appears to be complex and varies among depots, but research efforts directed at elucidating sex steroid action would be beneficial in understanding body fat distribution and implications on adult WAT homeostasis and energy balance.

### The Role of Estrogen in Regulating WAT Depot APCs

The observation that ovariectomy—increases WAT mass whereas estrogen replacement therapy decreases it—demonstrated that estrogen could significantly control adiposity. However, what are the cellular and molecular mechanisms determining these outcomes? While studies on adipocytes have yielded compelling evidence that estrogen-ER can regulate lipid storage and lipolysis, recent efforts have focused on investigating the role of estrogen-mediated APC kinetics to control adipocyte number and WAT health. Estrogen has been shown to stimulate APC proliferation, specifically within subcutaneous fat (68, 227). Moreover, there appears to be an intrinsic proliferative difference between female and male APCs. For instance, an observational study examining non-obese men and women revealed a 10% and 33% increase in the appearance of preadipocytes within the stromal vascular fraction of women’s abdominal and femoral subcutaneous fat, respectively (228). Further complicating this notion is the appreciation of inter-depot-specific effects on APC proliferation and differentiation determined by the WAT microenvironment (229). Transplantation studies have established functional differences between visceral and subcutaneous WAT APC function (229). For example, transplanting visceral APCs into subcutaneous depots changes the characteristics of this depot towards visceral WAT and impairs metabolic function (230). In reverse, transplanting subcutaneous APCs into visceral fat changes the visceral fat depot towards subcutaneous characteristics while improving metabolic performance (231). While these studies are intriguing, it is still unclear how the WAT microenvironment can alter APC function in a sex-dependent and depot-specific. The answer to this question may be multifaceted. These effects on depot-specific APCs could be attributed to variations in WAT developmental origins, innervation, vascularization, adipokine profiles, immunological composition, and extracellular matrix arrangement, all of which could be influenced by sex steroids.

### The Role of Estrogens in Regulating WAT Adipogenesis

Sex steroids also appear to impact adipogenic potential; however, this appears to be less straightforward. Studies have shown that estrogen can inhibit and promote adipogenesis *in vitro* (132, 232, 233). These differences in adipogenic action in response to estrogen may reflect estradiol concentrations, timing, type of APC tested (subcutaneous vs. visceral APC), or cell line (3T3-L1). Interestingly, at high concentrations and exposure, the estrogen mimetic BPA has been suggested to promote adipogenesis; yet environmentally relevant concentrations have shown no impact on adipogenesis (234, 235). Moreover, these effects on adipogenesis may be secondary as estrogen can attenuate the conversion of cortisone to cortisol, blunting receptors that bind glucocorticoids, which can facilitate adipocyte differentiation (236). For example, changes in the conversion of cortisone to cortisol could suggest implications for the activation status of the mineralocorticoid receptor (MR). Aldosterone activates MR in epithelial tissues to control and regulate plasma volume, blood pressure, and fluid homeostasis (237, 238). Interestingly, MR has a 10-fold higher affinity for glucocorticoids than aldosterone but relies on converting cortisol to the inactive metabolite cortisone by 11β-hydroxysteroid dehydrogenase 2 (11β-HSD2) (239). This conversion by 11β-HSD2 guarantees aldosterone-MR activation, but if estrogens disrupt this conversion process, MR activation alters adipogenic potential and adipocyte function. Indeed, MR expression increases with adipogenesis and appears to drive Pparγ expression (237). Interestingly, obese subjects have higher MR expression in visceral WAT than subcutaneous WAT (240). In agreement, mice carrying an adipocyte-specific deletion of MR were protected from diet-induced obesity and had improved energy balance (241). Moreover, *in vitro* adipogenic assays demonstrated that adipose stromal cells lacking MR had impaired adipocyte differentiation but could be rescued by a Pparg agonist (241).

Only a few studies have assessed the effects of sex on *in vivo* adipogenic kinetics. Guertin and colleagues have shown that developmental subcutaneous and visceral adipocytes appear to arise from distinct progenitors in a sex-dependent manner (196). In agreement, Rodeheffer and colleagues have demonstrated sex-dependent WAT depot differences in APC proliferation and differentiation potential (124). Using adipose lineage tracing and deletion tools, Graff and colleagues demonstrated that ERα regulates adiposity by controlling APC proliferation, adipose lineage fate, and beige fat formation (68). It was found that the loss of ERα within the adipose lineage results in lipodystrophy due to cellular lineage fate switching from adipogenic to myofibrotic. While these studies inform the cellular regulation and requirements of ERα activity, it does not entirely demonstrate how estrogen signaling and transcriptional activity regulate APC fate, proliferation, or adipogenic potential. For that matter, it is unclear what the molecule regulators are that control ER activity in APCs or adipocytes. It would be critical to evaluate signaling and transcriptional networks that control ER availability to determine how estrogen alters APC kinetics and overall adipogenic potential. Elucidation of these pathways will provide essential insight and new inroads into the regulatory functions of estrogen receptor signaling in adipose tissue biology.

## Thermogenic Fat: Burnin’ for you

The evolutionary adaption of brown and beige adipose tissue was essential for mammalian survival in response to temperature fluctuations. Unlike white adipocytes, brown and beige adipocytes (thermogenic fat) are capable of futilely burning substrates to generate heat rather than chemical energy (242). To do so, these cells rely on specialized mitochondria that express uncoupling protein 1 (Ucp1), which can collapse the proton gradient, thereby “uncoupling” the electron transport chain (243). However, the ability to activate and/or recruit thermogenic fat requires exposure to cold temperatures (15°C humans) or the use of β3-adrenergic receptor agonists (244, 245). The latter appears to have contraindication in human health due to potential cardiovascular disorders and changes in blood pressure (246). Yet, because of this unique ability, thermogenic fat is clinically desirable and may possess anti-obesity and diabetic properties and has been the focus of intense research in the past decade. While brown and beige adipocyte share hallmarks of thermogenesis and are recruited and activated by cold temperature exposure they appear to emanate from different lineage. For example, brown adipocytes reside in distinct stereotypical locations throughout the body that originate from the muscle developmental lineage (247). In contrast, beige adipocytes are recruited within WAT upon cold temperature stimulation from a smooth muscle cell lineage (194, 248). Critically, adult humans can generate cold temperature induced thermogenic fat cells; however, it is debatable if they are brown or beige adipocytes (249, 250). Sex steroids have also been implicated in thermogenic fat development and thermogenic action. Graff and colleagues demonstrated that smooth muscle cells could generate beige fat cells but not brown fat (194). Further, they showed that the deletion of ERα within the adipose lineage results in a fate switch favoring smooth muscle cells that were accompanied by beige fat formation (68). Yet, in opposition, Clegg and colleagues demonstrated that the activation of ERα alpha promotes thermogenic fat cell formation within WATs (251). In agreement, studies in aromatase knockout mice demonstrated a reduction in brown adipose tissue (BAT), and this phenotype could be rescued in response to estradiol replenishment (100). As discussed above, ERα null mice have increased adiposity and have reduced energy expenditure which could be associated with a decline in mitochondria electron chain genes (252). However, the effects of estrogen on thermogenic fat and its activity may be indirect. For instance, sympathetic denervation of BAT drastically reduces the ability of estrogen to initiate thermogenesis (253). Another observation stemmed from ERα specific deletion within the medial amygdala neurons, which resulted in decreased energy expenditure and increased adiposity (254). An additional observation showed that melanocortin receptors are expressed along neuronal projections that can stimulate Ucp1 expression and may also be activated by estrogens (255, 256). However, it appears that estrogen’s role in thermogenesis may be more complex than initially hypothesized, and further studies aimed at specific tissues and cell types might be more informative. Because of the therapeutic ability of thermogenic fat, it will be critical to continue to evaluate the role of estrogen and ERs in controlling thermogenesis and energy expenditure.

## Conclusion

Obesity is a devastating global public health issue that is multifaceted, relying on multiple disciplines to counteract its adverse effects (4). Yet, central to the obesity problem is WAT expansion and accumulation; thus, understanding the influencing factors and molecules that can regulate fat growth is a clinical ideal. Unequivocally, men and women differ in body fat accumulation and distribution. Overall, studies have demonstrated that modulating sex steroid bioavailability controls body fat determination, specification, expansion, and distribution. Throughout this review, we have attempted to highlight various aspects and questions that we think need critical attention and resolution to develop how estrogen and ERs regulate WAT health. While recent efforts have begun to untangle some of these roadblocks, significant gaps in the identification of the cellular, molecular, and transcriptional mechanisms governing sex steroid action still exist (27, 37). What is clear is the notion that estrogen fosters subcutaneous fat growth and blunts visceral fat expansion both at the adipocyte and APC levels. Contributing to WAT metabolic health, estrogen blocks WAT hypertrophy, increases WAT vascularization, protects WAT immunological cell composition, prevents WAT fibrosis, and promotes healthy WAT adipokine expression profiles. In addition, recent evidence suggests that estrogen and ER activity regulate APC kinetics and adipogenic progression. However, with the recent identification of multiple adipogenic cellular pools, it will be critical to ascertain which aspects of the adipose lineage estrogen targets. For that matter it would be critical to identify factors regulating estrogen bioavailability and receptor expression and activation. Moreover, because estrogen can regulate vascular growth, how might this affect APC biology and WAT hyperplasia? These questions become diagnostically essential for modulating estrogen bioavailability and could suggest alternatives to HRT in postmenopausal women. Collectively, more research is needed to understand the biological roles and regulation of sex steroids and estrogen on adipose tissue biology to foster metabolic health.

## Authors Contributions

BMS wrote the draft and revised the review. DCB revised and wrote the final version. All authors contributed to the article and approved the submitted version.

## Funding

This work is supported by the NIDDK awards K01 DK109027, and R03 DK122193 to DCB.

## Conflict of Interest

The authors declare that the research was conducted in the absence of any commercial or financial relationships that could be construed as a potential conflict of interest.

## Publisher’s Note

All claims expressed in this article are solely those of the authors and do not necessarily represent those of their affiliated organizations, or those of the publisher, the editors and the reviewers. Any product that may be evaluated in this article, or claim that may be made by its manufacturer, is not guaranteed or endorsed by the publisher.
